# Traumatic stress and post-traumatic growth in individuals who have had Covid-19: The mediating effect of resilience and moderating effect of psychological flexibility

**DOI:** 10.1371/journal.pone.0310495

**Published:** 2024-12-30

**Authors:** Fulya Türk

**Affiliations:** Faculty of Education, Department of Guidance and Psychological Counseling, Yildiz Technical University, Istanbul, Türkiye; University of São Paulo, BRAZIL

## Abstract

The Covid-19 pandemic has had a global impact and has negatively affected the mental health of individuals. It is known that depression, anxiety and traumatic stress levels are high in individuals who have experienced Covid-19. In light of this, an increase in Post-Traumatic Growth (PTB) levels is expected. This study examines the role of resilience and psychological flexibility in the relationship between traumatic stress levels experienced in Covid-19 and PTG levels. A total of 845 (64.18% males, ages ranged from 19 and 73, with an average of 36.80 ± 8.43) adults from Türkiye participated in the online survey. Impact of Event Scale-Revised, The Posttraumatic Growth Inventory, The Psychological Resilience Scale, and Psychological Flexibility Scale were used as measurement tools. According to the research findings, a partial mediator role of resilience has emerged in the relationship between individuals’ traumatic stress levels and PTG levels. Additionally, the regulatory function of psychological flexibility was determined in the relationship between traumatic stress levels and PTG levels. Accordingly, the link between Covid-19-related stress and PTG becomes stronger as the individual’s psychological flexibility increases. Traumatic stress has a more positive effect on PTG in individuals who exhibit high psychological flexibility. Based on these results, it can be confirmed that resilience and psychological flexibility are crucial factors to maintain the spiritual well-being of individuals during the Covid-19 pandemic, a worldwide crisis. Therefore, they need to be considered in the field of preventive mental health.

## Introduction

The Covid-19 pandemic, which began in late 2019 and persisted for three years, has had a profound global impact, significantly increasing levels of depression, anxiety, and stress [1–3]. These mental health issues are particularly severe in individuals who contracted the virus, who exhibit higher levels of depression, anxiety, and PTSD compared to those who did not get infected [3,4]. The severity of Covid-19 and the necessity for hospitalization further amplify PTSD symptoms [5]. PTSD, which arises from experiencing traumatic events involving real or perceived threats to life or severe injury, leads to intense fear and helplessness, avoidance of trauma-related stimuli, and reduced general reactivity [6]. Given the high prevalence of PTSD in previous coronavirus outbreaks, a considerable proportion of Covid-19 survivors may develop PTSD [7]. Although the handling of the Covid-19 pandemic in the light of PTSD diagnostic criteria is a matter of debate [8,9], it is clear that this crisis with its global impact presents an extremely challenging experience for individuals. The uncertainty, psychological distress, and emotional burden of being a virus carrier contribute to significant distress among Covid-19 survivors [10].

### Post traumatic growth (PTG)

Post-traumatic growth (PTG) refers to the positive psychological changes that occur as a result of struggling with highly challenging life circumstances. Unlike developmental growth, PTG is specifically linked to significant life crises and emerges as an outcome rather than a coping mechanism [11]. PTG is related to PTSD, with moderate to high PTG levels observed in trauma survivors [12,13]. During the Covid-19 pandemic, evidence suggests an increase in PTG, with individuals reporting closer relationships, heightened self-awareness, and greater appreciation for life, family, and friends [14–17]. Various factors contribute to PTG development during the pandemic, including timely diagnosis, psychological and social support, personality traits, and frequency of Covid-19 news consumption [18]. Qualitative studies investigating the post-traumatic growth process of individuals experiencing Covid-19, in line with PTG’s theory, show that individuals experiencing Covid-19 experience positive changes such as coping strategies, existential growth, lessons learned from diseases, new opportunities and social growth [19] found that they appreciated survival more, set new goals, improved their relationships, and became more aware of their personal development and health [17]. Many factors may play a role in this important change in individuals. Understanding the relationship between PTSD and PTG during the pandemic is essential, with resilience potentially serving as a key protective factor.

### The role of resilience

Resilience is the capacity to achieve positive outcomes despite significant risks that threaten development and adaptability [20]. It is not merely a personality trait but is measured by an individual’s ability to adapt in high-risk conditions. Positive outcomes without such risks are considered successes rather than resilience [21]. Resilience involves three core components: risk factors, protective factors, and positive outcomes [22]. Distinct from PTG, which denotes a transformation in functioning, resilience is about enduring and recovering from high-stress conditions [11]. During the Covid-19 pandemic, resilience has been identified as a crucial factor for mental health, helping mitigate psychological distress [23,24]. Longitudinal studies confirm a positive relationship between resilience and PTG, highlighting the importance of fostering resilience to protect mental health and alleviate stress [25,26].

### The role of psychological flexibility

Psychological flexibility is the ability to engage with the present moment adaptively, confronting internal experiences such as emotions, thoughts, and bodily sensations without judgment or defense, and taking actions aligned with personal values [27]. It encompasses six interconnected processes: acceptance, cognitive diffusion, flexible present moment awareness, self as context, values, and committed action [28]. Individuals with high psychological flexibility are better equipped to use functional coping strategies in response to life challenges, crises, and traumatic events [29,30]. During the Covid-19 pandemic, psychological flexibility has been positively correlated with well-being and negatively correlated with depression and anxiety [31,32]. It has been found to mitigate Covid-19-related stress, enhance well-being, alleviate perceived stress and general anxiety or depression, and counteract the negative effects of health anxiety on mental health [33–35]. Enhancing psychological flexibility is essential to reduce or prevent negative psychological impacts during the pandemic, and understanding its role in trauma stress and PTG levels can help improve mental well-being.

### The present study

This study aims to investigate the mediating role of resilience in the relationship between traumatic stress and PTG among individuals who have experienced Covid-19. Additionally, it explores whether psychological flexibility moderates the mediating effect of resilience on this relationship. Previous research has examined the link between Covid-19-related stress and PTG. However, our study focuses on the role of resilience and psychological flexibility in the relationship between these two important variables. Understanding these dynamics can help develop strategies and policies to enhance individual well-being during future global crises and contribute to research involving a population directly affected by Covid-19.

H1. Resilience mediates the relationship between traumatic stress and PTG in individuals who have had experienced Covid-19.
H2. Psychological flexibility moderates the mediating effect of resilience on the relationship between traumatic stress and PTG.

## Method

### Participants and procedure

Participants (n  =  845) were general public who were infected with COVID-19 disease in Türkiye. Their age ranged between 19 and 73 (*M*  =  36.80, *SD*  =  8.43). Participants were predominantly males (64.18%), married (76.48%), university graduate (64.18%), belonged to average socioeconomic background (84.63%), unvaccinated (96.45%), and received COVID-19 treatment (74.94%) with no psychological support (97.16%). Most of them (40.66%) got over the disease at home with moderate to severe symptoms. They reported that they did not transmit the disease to their relatives (55.79%) and their relatives were not infected with the disease (93.50%). A detailed description of participants characteristics is presented in Table 1. Data were collected between July 2021 and October 2021, using convenience sampling. A web-based online data collection form was prepared and sent to the participants via a link. The form first showed a brief information about the research goal and possible risks, and contributions. In addition, informed consent was obtained from the participants. In the first question after the consent, the participants were asked about their Covid-19 status and if they did not have Covid-19, they did not continue the research.

**Table 1:** Participants’ characteristics.

Variable	Level	n	%
Gender	Females	303	35.82
	Males	543	64.18
Marital status	Married	647	76.48
	Single	199	23.52
Education level	Literate	2	0.24
	Primary school	12	1.42
	Secondary school	8	0.95
	High school	181	21.39
	Undergraduate	543	64.18
	Postgraduate	100	11.82
Economic level	Below average	89	10.52
	Average	716	84.63
	Above average	41	4.85
Status of COVID 19 vaccination	Yes	30	3.55
	No	816	96.45
COVID 19 treatment	Yes	634	74.94
	No	212	25.06
COVID 19 treatment process	I’ve got over it at home without showing any symptoms.	55	6.50
	I’ve got over it at home with mild symptoms.	313	37.00
	I’ve got over it at home with moderate to severe symptoms.	344	40.66
	I’ve got over it at home with severe symptoms with support from the hospital.	79	9.34
	I was hospitalized with severe symptoms.	49	5.79
	I had a very difficult time and was treated in intensive care.	6	0.70
Transmitting the disease to relatives	Yes	374	44.21
	No	472	55.79
Relatives infected with COVID 19	Yes	55	6.50
	No	791	93.50
Psychological support	Yes	24	2.84
	No	822	97.16
Having children	Yes	590	69.74
	No	256	30.26
Number of children	No children	6	0.71
	1	206	24.35
	2	273	32.27
	3	87	10.28
	4 and more	19	2.25
	No response	255	30.14

### Measures

#### Traumatic stress

The Impact of Event Scale-Revised (IES-R) was utilized to assess the stress experienced by individuals during the Covid-19 transmission process [36]. The Turkish adaptation of the scale was conducted by Çorapçıoğlu et al. (2006) [37]. This scale comprises 22 items (e.g., "Everything similar brings to mind and reminds me of my feelings about the event." "I try not to think about the event.") rated on a scale from 0 (Not at all) to 4 (Very much). The internal consistency coefficient of the Turkish version is α = 0.94. For this study, the internal consistency coefficient is provided in Table 2. Participants were instructed to respond based on their experiences during their Covid-19 infection period.

**Table 2:** Means, standard deviations, and correlations with confidence intervals.

	Descriptive		Reliability			Correlation		
Variable	*M*	*SD*	Skewness	Kurtosis	α	1	2	3
1. Impact of event	34.09	18.33	0.16	-0.63	0.94			
					0.96			
2. Posttraumatic growth	55.11	25.40	-0.41	-0.71		.33**		
						[.26, .39]		
3. Psychological resilience	123.31	18.52	-0.08	-0.65	0.89	-.31**	.10**	
						[-.37, -.24]	[.03, .16]	
4. Psychological flexibility	129.40	15.02	0.23	0.24	0.70	-.43**	-.02	.60**
						[-.49, -.38]	[-.08, .05]	[.56, .64]

*Note*. *M* and *SD* are used to represent mean and standard deviation, respectively. Values in square brackets indicate the 95% confidence interval for each correlation. The confidence interval is a plausible range of population correlations that could have caused the sample correlation (Cumming, 2014).

* indicates *p* < .05.

** indicates *p* < .01.

#### Posttraumatic growth

The Post-Traumatic Growth Inventory was used to measure post-traumatic growth levels [38]. The Turkish adaptation was carried out by Kağan et al. (2012) [39]. The inventory includes 21 items (e.g., "The order of prioritization of the things I prioritize in life has changed." "I can accept things more as they are.") rated on a scale from 0 (I have not experienced this change at all as a result of stressful events) to 5 (I have experienced this change to a great extent as a result of stressful event(s)). The Turkish adaptation, consisting of three dimensions, has internal consistency coefficients of α = 0.88 for Change in Self-Concept, α = 0.78 for Change in Philosophy of Life, α = 0.77 for Change in Relationships, and α = 0.92 for the overall scale [39]. The internal consistency coefficient for this study is provided in Table 2.

#### Psychological resilience

The Psychological Resilience Scale for Adults was used to assess psychological resilience levels [40]. The Turkish adaptation was conducted by Basım and Çetin (2011) [41]. The scale comprises 33 items, rated on a five-point scale, with items presenting positive and negative characteristics to avoid biased responses. Participants responded to unfinished statements such as "When an unexpected event happens. . ." with options like "I always find a solution" or "Most of the time I do not know what to do." The test-retest reliability of the Turkish adaptation ranged from 0.68 to 0.81. The internal consistency coefficient for this study is provided in Table 2.

#### Psychological flexibility

The Psychological Flexibility Scale was employed to measure psychological flexibility levels [42]. The Turkish adaptation was carried out by Karakuş and Akbay (2020) [43]. This scale consists of 28 items (e.g., "I know what is important to me and where I want to be in my life") rated from 1 (Strongly disagree) to 7 (Strongly agree). The Turkish adaptation, comprising five dimensions, demonstrated good fit values and a reliability coefficient of α = 0.79 [43]. The internal consistency coefficient for this study is provided in Table 2.

#### Data analysis

Following initial analysis testing normality assumptions, and descriptive statistics mean, standard deviation, skewness and kurtosis values, Pearson correlation coefficient was computed to explore the correlations among variables. To test our main hypotheses, the PROCESS macro version 3.5 (Model 5—moderated mediation model) was used with a bootstrapping approach (10,000 resamples to estimate the 95% confidence intervals) for the significance of indirect effect [44,45] and mediation test. All data were analysed using SPSS version 24 and RStudio version 4.4.1. Assumption of univariate normality was checked (see Table 2) using skewness and kurtosis statistics by considering the threshold value of <|1.5| that suggests an acceptable level for a normal distribution [46].

#### Ethics

This study was conducted by the Declaration of Helsinki and approved by the Educational Sciences Ethics Committee of İstanbul Medeniyet University (REF = 2021/07-04). The data collection process of this research was carried out between 07/07/2021 and 07/11/2021. All participants gave informed consent and were informed that they could withdraw from the study at any time. Consent was obtained in writing from the individuals participating in the study. Accordingly, the individual was asked to give consent after reading the purpose, content and informed consent. The consent box was opened and asked to be checked. The ethics committee was informed about how to obtain the consent of the participants in the research conducted online and the approval of the ethics committee was obtained.

## Results

### Descriptive analyses and correlations

Prior to the main analysis, descriptive statistics, findings of correlation analysis, and internal consistency reliability estimates for each of variable are reported in Table 2. Descriptive statistics indicated that skewness and kurtosis values fell within a range of good normal distribution (skewness range = –.41 and .23; kurtosis range = -.71 and .24). The internal consistency reliability values for the scales of the current study ranged from acceptable (α = .70) to strong (α = .96). When it comes to the correlations between the variables, the results showed that impact of event had a positive and significant correlation with posttraumatic growth and that it had negative and significant correlations with psychological resilience and psychological flexibility. Also, posttraumatic growth had a significant and positive correlation with psychological resilience. Furthermore, there was a significant and positive correlation between psychological resilience and psychological flexibility as presented in Table 2.

### Testing moderated mediation hypothesis

**Fig 1:**
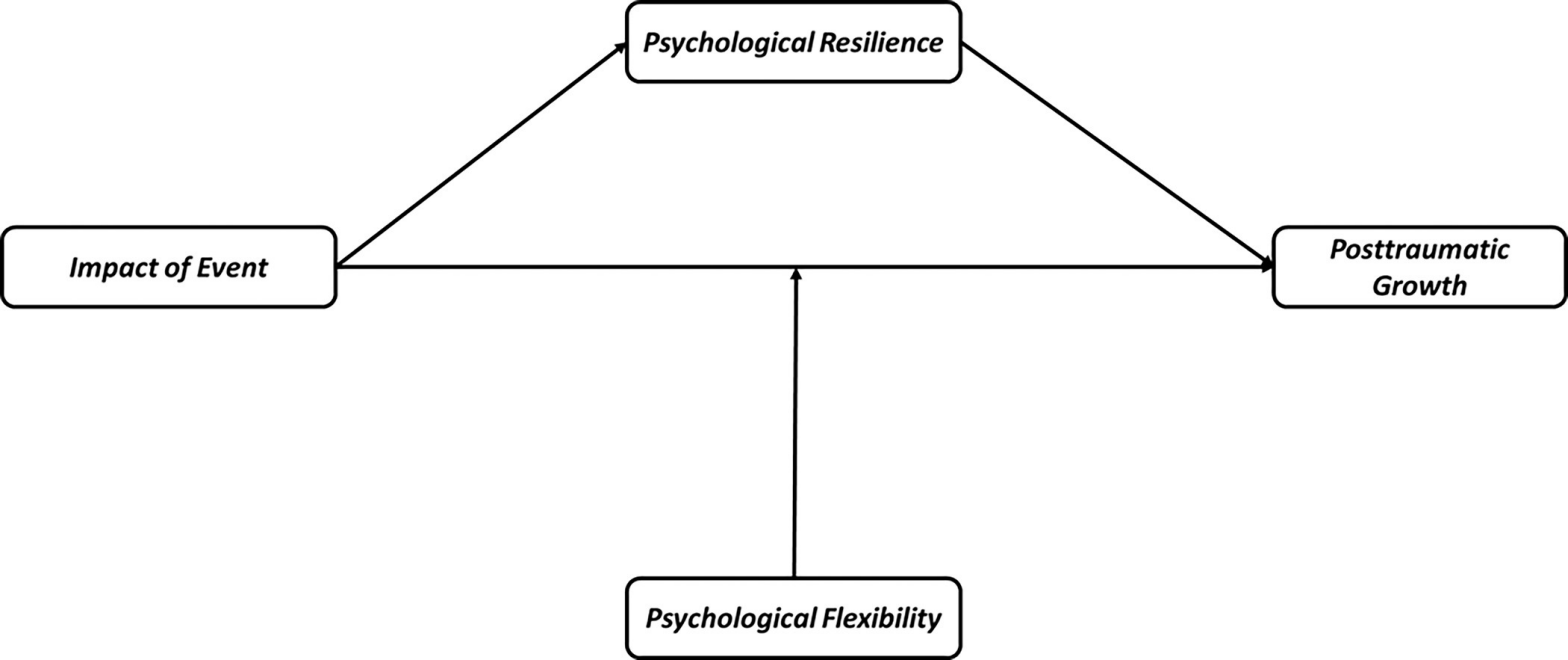
Moderated mediation model showing the associations between the variables.

**Fig 2:**
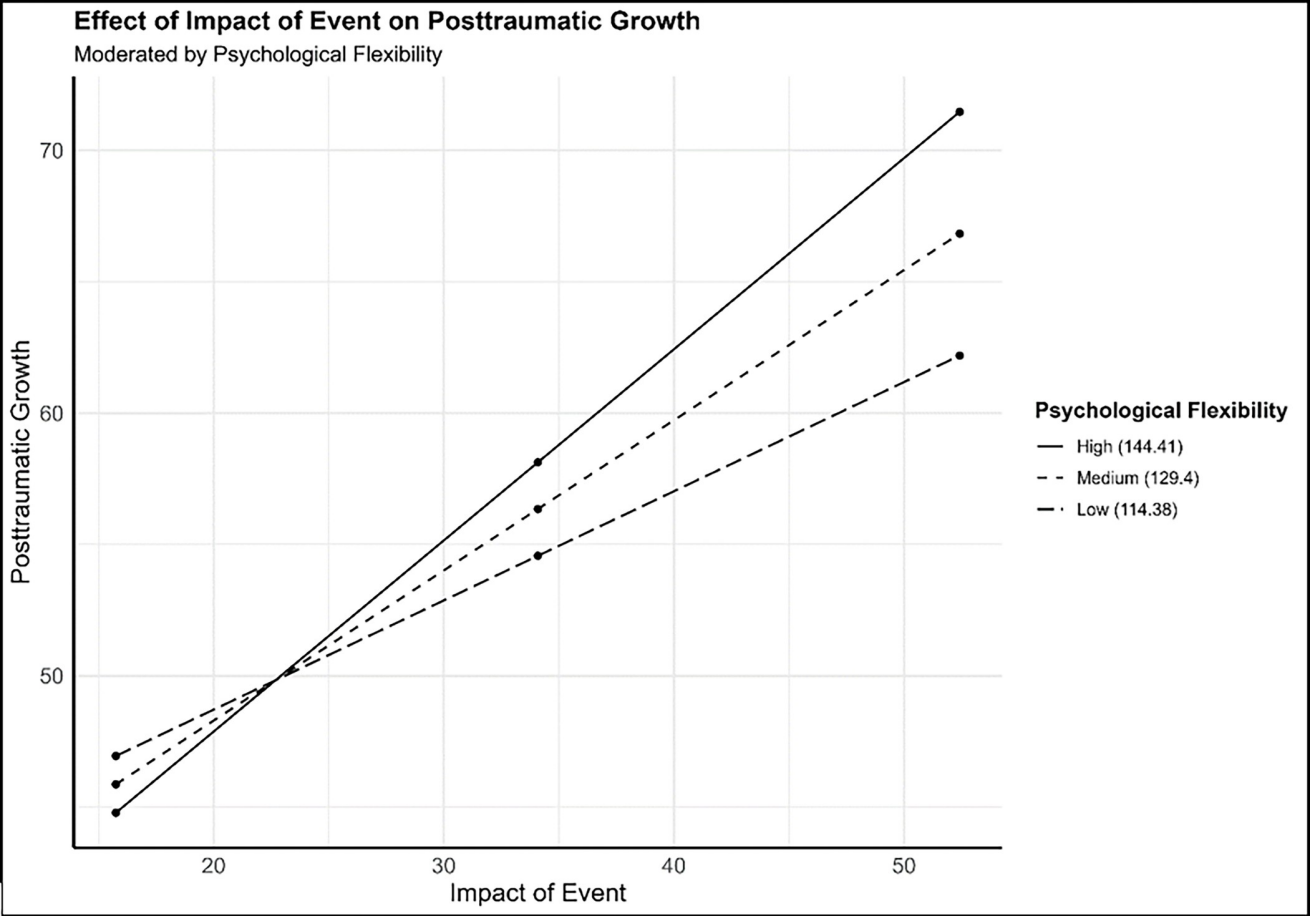
The simple slope indicating the moderation effects.

We tested model given in Fig 1, mediation with moderated direct path, model type was Model 5 based on Hayes Process models. It included two hypotheses, psychological flexibility moderates the relationship between impact of event and posttraumatic growth, and psychological resilience mediates the relationship between impact of event and posttraumatic growth. In moderation analysis (see Table 3), we found that interaction of impact of event and psychological flexibility, β = 0.01, SE = 0.003, *t* = 3.51, *p* < .001, 95% CI [0.005, 0.016], this regression model explained 16% of variance in posttraumatic growth R^2^ = .16, F(4, 841) = 40.86, *p* < .001, and interaction of variables changed variance significantly, ΔR^2^ = .012, F(1, 841) = 12.30, *p* < .001. As presented in Fig 2, the simple slope analysis indicated that the conditional effect of impact of event on posttraumatic growth occurred when psychological flexibility was low (-1 *SD*) β = 0.42, SE = 0.06, *t* = 6.76, *p* < .001, 95% CI [0.30, 0.54], moderate (0 *SD*) β = 0.57, SE = 0.05, *t* = 11.67, *p* < .001, 95% CI [0.47, 0.66], and high (+1 *SD*) β = 0.72, SE = 0.07, *t* = 10.74, *p* < .001, 95% CI [0.59, 0.86]. This indicates that a higher level of psychological flexibility, increases prediction effects of impact of event on posttraumatic growth.

**Table 3:** Unstandardized coefficients for the moderated mediation model.

	Consequent			
	*M* (Resilience)			
Antecedent	Coeff.	*SE*	*t*	*p*
*X* (Impact of event)	–.31	.03	–9.35	< .001
Constant	133.86	.03	104.51	< .001
	*R*^2^ = .09 *F* = 87.42; *p* < .001			
	*Y* (Posttraumatic growth)			
*X* (Impact of event)	-.77	.38	-2.02	.04
*M* (Resilience)	.26	.05	4.68	.00
*W* (Psychological flexibility)	-.24	.11	-2.11	.04
X*W	.01	.00	3.51	.00
Constant	35.89	14.82	2.42	.02
	*R*^2^ = .16 *F* = 40.86; *p* < .001			
Conditional indirect effects of impact of event on posttraumatic growth				
Psychological flexibility	Effect	BootSE	BootLLCI	BootULCI
*M* − 1*SD* (–15.02)	.42	.06	.29	.54
*M* (.00)	.57	.05	.48	.67
*M* + 1*SD* ((15.02)	.73	.07	.59	.59

We assessed the mediating role of psychological resilience on the relationship between impact of event and posttraumatic growth. The results of mediation analysis showed that impact of event significantly predicted resilience (β = –.31, *p* < .001), and impact of event (β = -.77, *p* < .001) and resilience (β = .26, *p* < .001) significantly predicted posttraumatic growth. Collectively, these two predictors explained 16% of the variance in posttraumatic growth.

The results revealed a significant indirect effect of impact of event on posttraumatic growth through resilience (b = -0.08, *SE* = .02, t = -3.96, 95% CI [-0.12, -0.04]), supporting meditation hypothesis. Furthermore, the direct effect of impact of event on posttraumatic growth in presence of the mediators was also found significant (b = 0.57, SE = .049, *t* = 11.67, *p* < 0.001). Hence, resilience partially mediated the relationship between impact of event and posttraumatic growth.

## Discussion

This study explores the roles of resilience and psychological flexibility in the relationship between traumatic stress symptoms and post-traumatic growth (PTG) among individuals who have experienced Covid-19. While previous research has examined the link between Covid-19-related stress and PTG, this study uniquely focuses on how resilience and psychological flexibility influence this relationship in individuals who have experienced Covid-19. The findings demonstrate that: (1) traumatic stress levels in individuals who have had Covid-19 predict PTG both directly and indirectly through resilience, and (2) the direct and indirect effects of traumatic stress on PTG vary based on individuals’ levels of psychological flexibility. Specifically, higher levels of psychological resilience enable individuals to exhibit fewer symptoms of stress and grow more after trauma.

The study reveals a positive and significant correlation between the impact of the traumatic event and PTG, alongside negative and significant correlations with psychological resilience and psychological flexibility. Additionally, PTG shows a significant and positive correlation with psychological resilience. Notably, there is also a significant and positive correlation between psychological resilience and psychological flexibility. Previous research indicates that individuals affected by Covid-19 often experience high levels of depression, anxiety, and PTSD [47], and there is a positive relationship between PTSD and PTG [48]. Furthermore, individuals with lower levels of resilience tend to exhibit more psychological symptoms [49], while social support and resilience function as protective factors [50], and there is a positive correlation between psychological resilience and PTG [51].

The primary aim of this study was to examine the mediating role of resilience in the relationship between traumatic stress levels and PTG. The findings indicate partial mediation by resilience, meaning that traumatic stress predicts PTG both directly and indirectly through resilience. This result aligns with previous research, which shows high levels of depression, anxiety, and PTSD among individuals affected by Covid-19 [47], a positive relationship between PTSD and PTG [48], and that individuals with lower resilience levels display more psychological symptoms [49].

Additionally, resilience has been shown to mediate the relationship between personality and psychological functioning during the pandemic [23], predict PTG [52], and play a crucial role in reducing the adverse psychological effects of the pandemic [53,54]. The reinforcing cycle between resilience and PTG over time further underscores the importance of resilience [14–25]. These findings support the conclusion that resilience enhances individuals’ ability to cope with Covid-19, rendering it a protective factor during this adverse period [55]. Indeed, when examining factors pertinent to PTG in the context of the Covid-19 pandemic, social support, stigmatization, and resilience emerge as crucial factors [56]. A meta-analysis on resilience highlights the profound impact of protective factors [57]. In summary, while "bouncing back" characterizes resilience, post-traumatic growth (PTG) can be seen as "bouncing forward" [58]. It can be posited that individuals may need to bounce back before they can leap forward, suggesting that post-traumatic individuals grow through resilience.

Psychological flexibility moderates the relationship between traumatic stress and PTG, with higher psychological flexibility amplifying the positive impact of stress on growth. This aligns with previous research suggesting that psychological flexibility can mitigate the negative effects of stress and support positive psychological outcomes [59,60]. High psychological flexibility enables individuals to adapt more effectively to stressful situations, leading to greater PTG.

Psychological flexibility has emerged as a crucial factor during the Covid-19 pandemic. Research shows that psychological inflexibility exacerbates the negative impact of Covid-19 risk factors [59], while high psychological flexibility is associated with lower levels of depression and anxiety [60]. Moreover, higher psychological flexibility correlates positively with post-traumatic growth (PTG) [61], and serves as a buffer between perceived stress and PTSD symptoms, reducing the negative effects over time [62]. Psychological flexibility also helps explain symptoms of depression, anxiety, and insomnia, positioning it as a resilience factor [63]. Additionally, a longitudinal study during the Covid-19 pandemic found that increases in psychological inflexibility over time predicted subsequent declines in mental health [64].

The findings of this study are consistent with the theoretical basis and previous research on psychological flexibility. The link between psychological flexibility and well-being is important for the protection of mental health. The effectiveness of ACT in increasing quality of life and well-being and reducing psychological symptoms is consistent with the results of this study [65]. This is because the relationship between traumatic stress and PTG strengthens as psychological flexibility increases.

In sum, individuals who exhibit high levels of psychological flexibility have an enhanced capacity to use functional coping strategies when dealing with challenges, crises, and traumatic events in their lives [29,30]. In the context of the pandemic, these individuals skillfully manage distressing emotions, experiences, memories, and impulses created by the Covid-19 pandemic and the transition process. They achieve this by creating mental space for acceptance, staying present, and distancing themselves from their thoughts [66]. Furthermore, by centering their actions around their values, they potentially alleviate symptoms of traumatic stress and promote positive psychological changes in the face of these challenging circumstances [67]. After the diagnosis of Covid-19, individuals expressed greater appreciation for life, established closer family and friendship relationships, developed more awareness about their personal development and health, and felt grateful for social support [17–68]. This perspective, indicative of PTG, may be related to psychological flexibility, values, and acting in line with values.

### Limitations

This study bears several limitations. Firstly, data collection was conducted through convenience sampling within an online environment. The sample composition was notably skewed towards men, university graduates, and individuals with a moderate economic status. Additionally, factors associated with Covid-19 disease could have influenced the outcomes of the study, as a significant proportion of participants experienced the disease at home with moderate to severe symptoms. Another limitation is that the study did not evaluate the effect of conditions such as being infected with Covid-19 more than once or having lost relatives to the pandemic on PTG. Secondly, the duration of the traumatic experience appears to hold significance in the relationship between PTSD and PTG [13]. Studies have explored the link between the elapsed time since trauma and PTG. This study did not consider the duration between the occurrence of Covid-19 and individuals’ experiences, which can be identified as a limitation. Thirdly, the study relies on self-report measures as its data source. Fourthly, the research design is cross-sectional. Therefore, there is a need for longitudinal studies to unveil the dynamic association between PTSD and PTG over time. Fifthly, this study did not delve into the influence of cognitions on PTG [69] and the distinction between real and illusory growth [16]. Future studies should conduct longitudinal investigations to explore the role of cognitions in PTG. Sixthly, previous studies have emphasized the importance of factors such as self-esteem, coping tendency, social support, self-efficacy, perceived impact of Covid-19, and self-stigma in influencing PTG [13,48,70]. This study did not examine other psychological variables that may affect PTG. Future research should investigate these factors to provide a more comprehensive understanding of PTG. Lastly, it’s imperative to exercise caution in interpreting these results. There exists a partial mediation of resilience between traumatic stress due to Covid-19 and PTG. PTG may not be solely contingent upon resilience and is not necessarily a prerequisite. Resilience might emerge either before or after trauma, whereas PTG signifies transformative changes experienced by an individual in response to traumatic events [14]. The observed partial mediation supports this perspective.

### Implications

The findings of this study underscore the significant roles of resilience and psychological flexibility in mitigating the mental health impacts of the Covid-19 pandemic and promoting post-traumatic growth (PTG). These insights offer several key clinical implications.

Given the important role of resilience in reducing the negative effects of traumatic stress and increasing post-traumatic stress, clinical interventions should include resilience training. Resilience-building techniques, such as stress management strategies, cognitive restructuring, and mindfulness practices, can be integrated into therapeutic programs for individuals recovering from Covid-19 and in other trauma situations.

This study highlights the importance of psychological flexibility between traumatic stress and PTG. Acceptance and Commitment Therapy (ACT), which focuses on increasing psychological flexibility through mindfulness and value-based actions, can be used as part of mental health interventions during and after the pandemic. It will be an important protective and preventive mental health service to provide therapeutic interventions aimed at improving psychological resilience and psychological flexibility, not only in cases of crisis and trauma, but also to the entire public, especially individuals at risk. Having these skills before individuals experience trauma and crises will reduce their psychological vulnerability.

Policymakers and researchers should prioritize funding and support for studies investigating resilience and psychological flexibility in diverse populations. This can inform the development of evidence-based policies and interventions that improve mental health outcomes during public health crises. By integrating these strategies into clinical practice, mental health professionals can more effectively support individuals in coping with the psychological challenges posed by the Covid-19 pandemic and enhancing growth and resilience in the face of adversity.

## Conclusion

Despite these limitations, this study offers important conclusions. This study is among the first to investigate the roles of resilience and psychological flexibility in the relationship between traumatic stress and PTG among individuals who have experienced Covid-19. The results suggest that both resilience and psychological flexibility play crucial roles in managing traumatic stress and fostering PTG. High resilience and psychological flexibility contribute to better coping and greater personal growth, highlighting the need for targeted interventions to support mental health during and after crises. Insights from this study can inform future research and public health strategies, providing valuable lessons for managing the psychological impacts of pandemics and other global challenges.

## Supporting information

S1 Data (XLSX)
